# How Can Synergism of Traditional Medicines Benefit from Network Pharmacology?

**DOI:** 10.3390/molecules22071135

**Published:** 2017-07-07

**Authors:** Haidan Yuan, Qianqian Ma, Heying Cui, Guancheng Liu, Xiaoyan Zhao, Wei Li, Guangchun Piao

**Affiliations:** 1 College of Pharmacy, Yanbian University; 977 Gongyuan Street, Yanji 133002, China; hdyuan@ybu.edu.cn (H.Y.); qianqian3918@163.com (Q.M.); 15504336519@163.com (G.L.); 2015010622@ybu.edu.cn (X.Z.); 2015010629@ybu.edu.cn (W.L.); 2 Key Laboratory of Natural Resources of Changbai Mountain & Functional Molecules, Yanbian University, Ministry of Education, Yanji 133002, China; 3 College of Traditional Chinese Medicine, Yanbian University; Yanji 133002, China; kimmy@ybu.edu.cn

**Keywords:** traditional medicines, network pharmacology, synergistic effects, drug discovery

## Abstract

Many prescriptions of traditional medicines (TMs), whose efficacy has been tested in clinical practice, have great therapeutic value and represent an excellent resource for drug discovery. Research into single compounds of TMs, such as artemisinin from *Artemisia annua* L., has achieved great success; however, it has become evident that a TM prescription (which frequently contains various herbs or other components) has a synergistic effect in effecting a cure or reducing toxicity. Network pharmacology targets biological networks and analyzes the links among drugs, targets, and diseases in those networks. Comprehensive, systematic research into network pharmacology is consistent with the perspective of holisticity, which is a main characteristic of many TMs. By means of network pharmacology, research has demonstrated that many a TM show a synergistic effect by acting at different levels on multiple targets and pathways. This approach effectively bridges the gap between modern medicine and TM, and it greatly facilitates studies into the synergistic actions of TMs. There are different kinds of synergistic effects with TMs, such as synergy among herbs, effective parts, and pure compounds; however, for various reasons, new drug discovery should at present focus on synergy among pure compounds.

## 1. Introduction

Although traditional medicines (TMs) are often underestimated, they have long been used to maintain health and to treat and prevent many human diseases [1,2]. Even today, despite advances in culture and medical understanding and access to general medical treatment, TMs continue to be widely used and play an important role in many countries; indeed, demand for TMs is increasing around the world. Historically, various cultures and civilizations have encouraged the large-scale practice of different TMs [3,4,5,6,7,8]. To fulfill its mission of saving lives and promoting health, the World Health Organization supports clinical research projects related to the safety and efficacy of TMs; it promotes strategic research into TMs and supports the ongoing development of such medicines [9]. Considerable evidence indicates that many effective, widely used drugs obtained from plants were originally based on TM applications [10]. Modern science has greatly improved the accessibility, acceptability, and convenience of TMs [11].

Artemisinin is an antimalarial drug extracted from *Artemisia annua*, which is an herb used as a traditional Chinese medicine for its antimalarial action. Artemisinin is a classic example of a modern drug developed from a TM. TMs also give rise to a great number of drugs, such as bicyclol, that do not follow the original actions of the TMs [12,13]. In the effort to discover new drugs, the existence of TMs cannot be ignored. Recent studies of traditional Chinese medicine (TCM) found that more than 12,000 species of plants and animals and tens of thousands of prescriptions are used in the practice of TCM [14,15]. By contrast, around the world, many health professionals, decision makers in public health, and the general public people doubt the security, efficacy, quality, and administrative supervision of TMs [9]. Even though clinical practice with TMs has existed for several thousands of years, such medicines exert adverse effects on patients if improperly applied. Some TMs from herbs that give rise to clinical side effects have been confirmed using pharmacological methods. For example, the compound podophyllotoxin may result in hepatotoxic injury; a few cases of thrombocytopenia have been reportedly induced by *Dysosma versipellis*; and aristolochic acid contained in *Aristolochia fangchi* may cause renal failure [16,17,18].

After thousands of years of development, TMs are currently facing severe challenges. To cope with the new difficulties facing TMs, various efforts are currently being undertaken. On 25 December 2016, the first law on TCM in China was passed by the country’s top legislature, and the law will take effect on 1 July 2017. The law is a milestone in the development of TCM: It clarifies the important position of TCM in China’s health-care system, and it encourages the development of such medicine. Of greater importance is that additional policies and regulations will be introduced to bring about the modernization of TCM (http://www.satcm.gov.cn; http://www.chinadaily.com.cn). The modernization of TCM, which aims to improve the practice of TCM in China, combines the advantages and characteristics of TCM with advanced science and technology. The purpose is to produce modern TCM products, which are in line with the standards and norms of international medicine and industry (such as Good Agricultural Practice (GAP), Good Laboratory Practice (GLP), Good Clinical Practice (GCP), Good Manufacturing Practice (GMP)). In this way, the further development of TCM will adapt to the demands of modern social development. At the same time, modernized TCM will achieve three types of efficiency (high efficiency, quick efficiency, and long-lasting efficiency) and three areas of “small” advantages (small dosage, low toxicity, and fewer adverse reactions). China has accumulated a wealth of experience in TCM, which will provide valuable support for its modernization [19,20].

In recent years, following the development of science and technology, such areas as molecular biology, metabolism, breeding and transgenics, compound separation and analysis are gradually being applied to the research and production of TCM. More in-depth basic research into TCM will be conducted [21]. In various aspects related to TCM, such as resources, research and development of new drugs, and clinical application, great volumes of research data have accumulated. The arrival of the era of big data has provided ways of thinking about how to achieve the modernization of TCM and how to develop new drugs inspired by TCM [22]. Both Western drugs and TMs have been under supervision of the Adverse Drug Reaction monitoring system in China since 1989, which aims to control any risks associated with the use of TCM through effective monitoring [23]. Of course, in China and other countries, more effective pharmacovigilance is essential for collecting and analyzing reliable information about the safe use of herbal medicines for their further development [24].

## 2. Synergistic Effects in Traditional Medicines

A TM prescription frequently contains different herbs or other components, and these have a synergistic effect in effecting a cure or reducing toxicity. Research into the synergistic effect among medicinal herbs and other components is ongoing. Different components in a prescription exert a synergistic effect in such ways as acting on different targets or improving the solubility of active compounds, which constitute the pharmaceutical basis of TMs.

Studies into TMs often focus on a single compound or single target, which has led to the identification of many effective components. However, in many cases, the efficacy of TMs is often the synergistic result of multiple components, targets, and pathways [25]. In many cases, with TM, a single compound extracted and separated from the active part of a prescription, shows much lower activity than the original prescription; in other cases, the single compound may be much more toxic. There is thus a big difference between compound and formula, and the synergistic effect of TMs has to be considered. It is necessary to clarify the synergistic effect of components and their pharmacological mechanisms. The synergistic effect of mixed drugs and herbal prescriptions has been studied in clinical applications. Multidrug methods have been used to treat such complex diseases as cancer, diabetes, and hypertension. One well-known case is the common use of a cocktail of drugs in synergistically treating HIV infection [26,27].

With TCM, tens of thousands of prescriptions have been recorded. To boost their therapeutic efficacy and reduce side effects, the prescriptions of TCM practitioners, based on their clinical experience, often involve mixtures of medicinal plant species and minerals. TM theory emphasizes maintaining a state of equilibrium among the various systems and functions of a person to promote health. Therefore, TCM prescriptions, in which each medicine devotes its own particular function to create a combined effect for treating diseases, have long been used in China [6,8]. TMs are able to combat disease because they contain active substances. A TM prescription often has many ingredients, which produce a synergistic effect by acting at different levels on multiple targets. Among the many ingredients in a TM, only those ingredients that exert a major effect can be termed active ingredients. The most important task in the modernization of TCM is to use modern scientific methods to clarify the mechanisms of these active ingredients [19].

**Figure 1:**
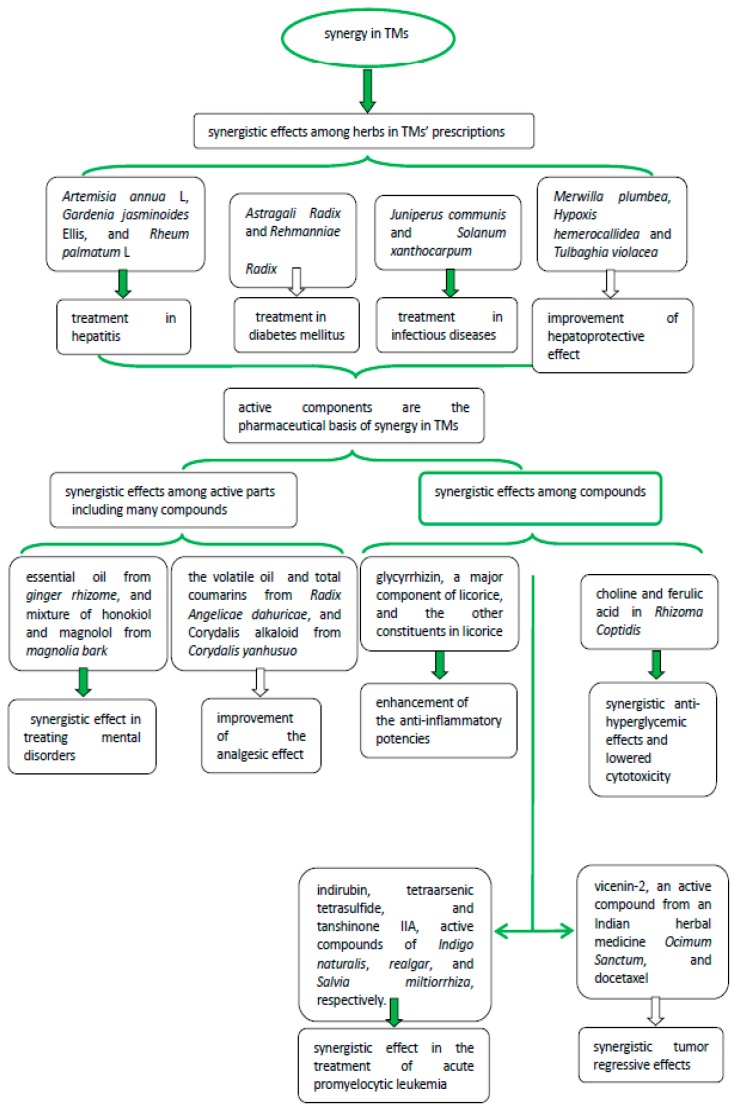
Synergistic effects in traditional medicines (TMs).

Emerging clinical studies on TCM have provided convincing evidence that has boosted its credibility and reputation outside China, such as artemisinin from *Artemisia annua* L. [28]. However, there are different kinds of synergistic effects in TMs, such as synergy among herbs, among effective parts, and among pure compounds. Multiple components of medicinal herbs offer great potential for synergistic actions. In recent years, it has become evident that synergistic effects of herbal combinations can be achieved by acting on multiple targets, reducing the antibiotic resistance of bacteria, diminishing side effects, and improving bioavailability in crude herb extracts [16]. Some synergistic effects in TMs are summarized in Figure 1.

### 2.1. Synergistic Effects among Herbs or Other Components in TM Prescriptions

The TCM prescription yin-chen-hao-tang (YCHT) consists of three Chinese herbs, and it has been used to treat various types of hepatitis. To identify the therapeutic effect of YCHT, combination therapy of the three herbs was investigated in a rat model of liver injury to determine the efficacy of each component. The study found that compared with the single components or partial combinations, combination therapy reduced significantly the total body clearance and markedly increased the maximal concentration and area under the concentration-time curve. In another study on YCHT using a liver injury rat model, combination therapy slowed the elimination rate, increased the bioavailability, and showed a stronger therapeutic effect than any one or two of the three components by acting on multiple targets [16,29]. Two Chinese herbs, rehmanniae radix and astragali radix, are extensively used to treat diabetes mellitus. In a rat model of foot ulcer, no one of the two herbs was able to reduce the diabetic wound area. However, when the two herbs were combined in a 2:1 ratio, they were clearly able to reduce the diabetic wound area compared with controls. That ratio may result in special effects on different targets, thereby producing a synergistic effect. However, it could be that the synergistic effect was produced as a result of new compounds created during the preparation of the herbs [16,30].

In South Africa, three traditional medicinal plants, *Merwilla plumbea*, *Hypoxis hemerocallidea*, and *Tulbaghia violacea*, are used to treat some infectious diseases. Using a microdilution method, the antimicrobial activities of leaf and bulb extracts of the three medicinal herbs (singly and in combination) were comparatively evaluated against *Candida albicans*, two Gram-negative bacteria, and two Gram-positive bacteria. It was found that the proportional combination of dichloromethane and petroleum ether extracts in the *Merwilla plumbea* bulb showed the strongest synergistic effect against *Staphylococcus aureus* [31]. Combined lower doses of *Juniperus communis* and *Solanum xanthocarpum* markedly improved the hepatoprotective effect compared with the individual effect of the two against azithromycin- and paracetamol-induced hepatotoxicity in rats. It was further found that a prescription containing multiple plant products had synergistic effects and could achieve the desired pharmaceutical result [32].

### 2.2. Synergistic Effects among Effective Parts

A combination of ginger rhizome and magnolia bark is used to treat mental disorders in many TCM prescriptions. Polysaccharides (PGR) and an essential oil (OGR) from ginger rhizome and a mixture of honokiol and magnolol (HMM) and polysaccharides (PMB) from *magnolia bark* were used alone and in combination to assess antidepressant effects in the mouse tail suspension test and forced swimming test. Co-administration of HMM with OGR produced a synergistic effect after two-week treatment; a synergistic increase in noradrenaline in the prefrontal cortex and significant increase in serotonin were observed. When OGR was administered together with HMM, they were able to produce synergistic effects by reducing functional abnormalities in the noradrenergic and serotonergic systems [33].

Using three methods, such as the DPPH free radical scavenging assay, the antioxidant activities of fractions from *Astragalus membranaceus* and *Glycyrrhiza uralensis* and combinations of the two were assessed in vitro. It was found that compared with the sum of the two single herbs the ethyl acetate extract of the pair of herbs showed a higher antioxidant ability; that extract contained a higher total phenolic and flavonoid content. Compared with the single herbs, the ethyl acetate extract exhibited a significantly higher antioxidant effect, higher activity of antioxidant enzymes, and better cytoprotection. The phenolic and flavonoid compounds were possibly the most important ones in inducing the synergistic antioxidant effect and cytoprotective action [34].

*Radix Angelicae dahuricae* and *Corydalis yanhusuo* together form the yuan-hu-zhi-tong TCM prescription, which is used to relieve various pains, such as those associated with gastralgia and dysmenorrhea. Corydalis alkaloid (CA, derived from *Corydalis yanhusuo*) has analgesic effects; the volatile oil (VO) and total coumarins (Cou) from radix *Angelicae dahuricae* both improve the analgesic effect of CA. In rats, CA has been found to weaken nociception produced by intraperitoneal injection of acetic acid in a dose-dependent manner; a close relationship was observed between the plasma concentration of dl-THP and the analgesic effect of CA. After oral administration of CA, CA-VO, CA-Cou, and CA-VO-Cou, the dl-THP levels in rats’ plasma were examined at different times. It was found that the *radix Angelica dahuricae* extracts improved the plasma concentration of dl-THP, thereby reinforcing the analgesic effects of CA [35]. *Echinacea purpurea* extracts are applied to treat and prevent upper respiratory infections. In vitro experiments showed that ethanolic *Echinacea purpurea* herba and radix extracts exerted synergistic pharmacological effects on the endocannabinoid system [36].

### 2.3. Synergism among Compounds

Glycyrrhizin (GC) is a major active component of licorice, and it is one of the most important herbs used in oriental TMs. An immunoaffinity column was combined with an anti-GC monoclonal antibody to remove 99.5% of GC from licorice extract (LE); the GC-removed extract (GC-KO extract) from LE was then tested. It was found that LE suppressed expression of inducible nitric oxide synthase (iNOS) and nitric oxide (NO) in lipopolysaccharide-induced RAW264 murine macrophage cells. However, the inhibitory effect on expression of iNOS and NO by treatment with GC or GC-KO extract alone was markedly less than that with LE. Notably, the weakened inhibitive effect could be improved by treating with a combination of GC and GC-KO extract. It was evident that when GC is used with the other components in LE, significant synergistic effects on suppression of iNOS expression may occur [37]. Co-administration of the main alkaloids and minor constituents, such as choline and ferulic acid, in *Rhizoma coptidis* showed lower cytotoxicity and synergistic anti-hyperglycemic effects than *Rhizoma coptidis* extract in vitro and in vivo [38].

Vicenin-2 is an active compound separated from an Indian herbal medicine, *Ocimum sanctum*. Potential synergistic tumor regressive effects were investigated using oral co-administration of vicenin-2 and docetaxel (DTL). The combined DTL and vicenin-2 treatment greatly reduced the expression of the proliferation marker Ki67 and angiogenic marker CD31. However, the co-treatment remarkably increased the expression of tumor suppressor E-cadherin compared with treatment by either of the two compounds [39]. Realgar-*Indigo naturalis* formula (RIF) is very effective in the treatment of acute promyelocytic leukemia (APL). *Indigo naturalis*, realgar, and *Salvia miltiorrhiza* are the main components of RIF; indirubin, tetraarsenic tetrasulfide, and tanshinone IIA are their active ingredients, respectively. A combination of the three active ingredients showed a synergistic effect in inducing APL cell differentiation in vitro and in treating a murine APL model in vivo. It was evident that tetraarsenic tetrasulfide was the principal component among the three active ingredients, whereas tanshinone IIA and indirubin had an ancillary function [40].

Further research into the synergy of TMs should proceed in step-by-step fashion to obtain a comprehensive understanding of those medicines for the greater benefit of humankind. Collaborative studies face many difficulties, so help in this direction is urgently needed, especially in terms of theory and methodology.

## 3. Network Pharmacology

Although the one-drug, one-target development model has achieved great success, its limitations have become evident. Many different drugs can act on the same single-drug target, and most drugs have more than one target. That is particularly the case with complex diseases, such as neurological disorders, cancer, and cardiovascular diseases. Those diseases are pathologically related to the interacting outcomes of multiple pathways, multiple genes, and multiple functional proteins. For example, cancers are progressively being accepted as systemic diseases that result from the interaction of multiple biological networks of pathways, genes, and proteins. Accordingly, in many cases, cancer cannot be eradicated by therapy that targets a single pathway, gene, or protein. By contrast, the pharmacological actions of drugs with multiple targets have better curative effects [41,42,43].

If a disease alters the complex equilibrium of the human body, recovery of the original equilibrium is a promising target in drug design. The human biological system is complex and robust. The structure and function of that system remains stable despite internal and external damaging forces. It is difficult to break the dynamic balance of the human biological system by perturbing only one or a few targets. Drugs often need to adjust to multiple targets, and here network pharmacology constitutes a dramatic development. Network pharmacology develops molecular drug targets and molecular targets; it targets biological networks and analyzes the links among drugs, targets, and diseases in those networks; the underlying mechanisms of the drugs it uses demolish the biological networks of disease [25,44,45]. In network pharmacology, the focus is on the balance network (or robustness) and perturbations in that network; the emphasis is on the biological status of drug effects and the spectrum of their dynamics instead of examining just the role of a single target or a few “fragmented” targets [46].

Thus, changes are taking place in pharmacology. Pharmacotherapeutic strategies with central nervous system (CNS) pathologies are shifting from targeting individual disease-causing genes to targeting disease-causing networks—even though most CNS-acting drugs are initially designed to function on individual disease-causing genes. The discovery of anti-cancer drugs has used the network approach to achieve significant results [47,48,49]. Network pharmacology, which aims to study the complex, varied relationships among targets, drugs, diseases, and pathways, presents a new approach for drug discovery. To obtain better anti-tumor effects, optimized combinations of multiple drugs are often applied to synergistically attack different pathways. The use of multiple drugs has been demonstrated to be effective in resolving complex relations among various pathways related to cancer stem cells [42].

Network pharmacology has benefited considerably from the identification of new bioactive ingredients and clarifying the mechanisms of TM prescriptions. A case study was provided on a zhi-zi-da-huang decoction (ZZDHD), a classic TCM formula, which has been used to treat liver disease in clinical practice. The concrete procedures were as follows:

First, the structures of 30 compounds of ZZDHD, which were identified in previous study, were performed by MMFF94 Force Field in Discovery Studio 2.5(DS 2.5).

Second, the crystal structures of all candidate targets were collected from RCSB Protein Data Bank, and related proteins including CYP2E1, iNOS, etc., were selected as potential targets. All proteins were made optimal using the CHARMm Force Field in DS 2.5.

Third, Molecular docking was performed with the LibDock protocol according to the CHARMm Force Field in DS2.5.

Fourth, by utilizing Cytoscape 3.0.2, the drug–target network was built based on the top 10 of the molecular docking level.

Finally, the active compounds were predicted through molecular docking, and network analysis showed that multiple active pharmaceutical compounds of ZZDHD were found to act on different targets [50].

In this study, 30 compounds of ZZDHD identified by HPLC-PDA-ESI-MS/MS in previous work were collected for further research. However, identification like this is not enough: many compounds would be missing because of lower contents, and new compounds or some compounds with specific characteristic cannot be identified. Therefore, more detailed study concerning compounds of ZZDHD should be implemented to obtain more reliable results. Furthermore, because antioxidant effect is related with too many pharmaceutical effects, the results were not too persuasive to explain the effects of ZZDHD on alcoholic liver disease. Thus, the relevant targets should be more specific for alcoholic liver disease.

Furthermore, in another study, a new network-based inference (NBI) method, domain tuned-hybrid (DT-Hybrid), which develops a well-built recommendation technique by domain-based knowledge, has been fully examined using an already-proved drug-target interaction database obtained from DrugBank [51]. To find more active compounds and relevant mechanisms, comparisons between different NBI methods are further needed.

Holism is increasingly accepted in biological interpretations and predictions; consequently, the concept of network pharmacology is emerging in the discovery of new drugs. In networks, the basic unit is a node, which can be a disease, target, or a drug; the connection between any two nodes is termed an edge. A special group of related nodes is called a module; the connection between any two modules is termed a bridge. In addition, choke points, bottlenecks, hubs, degree, and betweenness are promising targets or parameters for drug discovery. The robustness of biological networks is the ability to withstand internal and external disturbances [43,52,53]. Network pharmacology is continuously developing. Visual network pharmacology has been developed and designed to visualize the complex relationships among diseases, targets, and drugs in network pharmacology research. The introduction of various techniques and methods, such as proteomics, can help scientists predict and understand the mechanisms underlying the multiple actions of drugs; such introduction can also uncover multiple drug targets for combined therapeutic solutions or for designing and optimizing multi-target drugs. In addition, high-throughput technologies are applied in network pharmacology to reveal and predict mechanisms by constructing drug-target-disease networks and analyzing large amounts of data [41,45,54].

**Figure 2:**
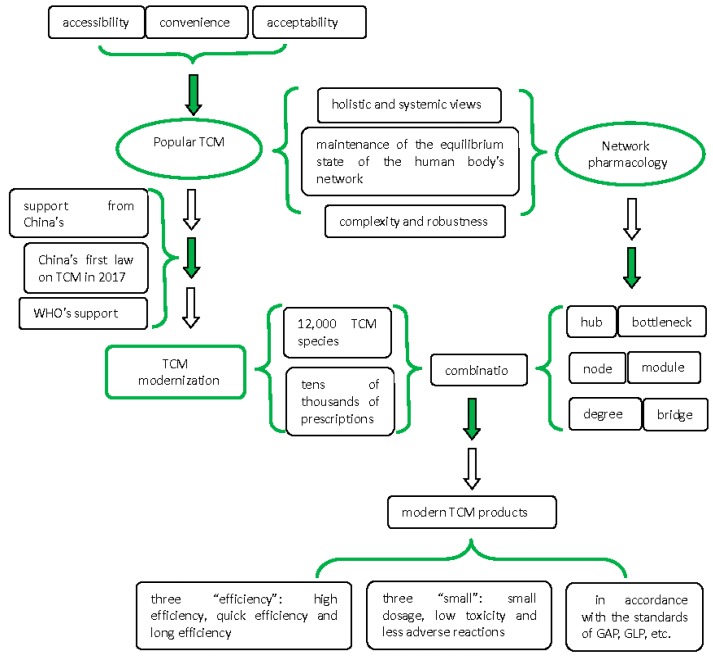
Traditional Chinese medicine (TCM) modernization and network pharmacology.

The systematic use of network pharmacology methods and approaches to drug discovery will increase clinical success rates. Many drugs often target the same protein; at the same time, one drug often functions on many different targets. Many pharmacological networks have been developed not only to clarify the mechanism of a single compound drug but also to probe the effects and underlying mechanisms of drug combinations. TCM formulas have multiple compounds, which function pharmacologically on biological networks, instead of single targets [55,56,57]. Based on a holistic and systemic approach, drug combinations with two or more compounds could have synergistic effects for complex diseases. Thus, network pharmacology will become the next paradigm in drug discovery. The connections or assumptions between TCM modernization and network pharmacology are shown in Figure 2.

## 4. Application of Network Pharmacology in TMs

Comprehensive, systematic research into network pharmacology is consistent with the view of holisticity, such as dynamic syndrome differentiation, and an overall comprehensive approach that is a main characteristic of many TMs, such as TCM [45,58]. In recent years, the network pharmacology method has been used to analyze the diseases and mechanisms of pharmacology in TCM. The herbal prescriptions of TMs have great therapeutic value and represent an excellent resource in drug discovery. Therefore, identifying the features of multiple components, multiple targets, and their actions and possible synergistic effects in TMs are a major challenge in research.

As a new scientific approach, analyzing network data and systems biology is the basis of network pharmacology. The combination of TCM and network pharmacology bridges the gap between modern and traditional medicine. The general analysis process of TM and network pharmacology involves retrieving interaction information to construct networks and analyzing and discovering findings from network models [52].

### 4.1. Research into TM Compounds Using Network Pharmacology

One study used the PharmMapper database to obtain information about the major active compounds in the si-wu decoction (a TCM prescription) and analogous prescriptions (including ligustilide, peoniflorin, and senkyunolide), and to construct compound–protein networks. The study used Cytoscape software and the Kyoto Encyclopedia of Genes and Genomes pathway database to construct and visualize the networks of compounds, targets, and pathways. It was found that 51 pathways were involved in the effects of the main active compounds and that serine/threonine protein kinases were the potential targets of the *si-wu* decoction and analogous prescriptions. Those results underscore how the prescriptions function use multiple compounds, pathways, and targets [59]. Another study on Chinese herbal medicines used the 3D structure database of Beijing University and a database of Chinese natural products: It identified 1323 small molecular compounds from clearing-heat herbs (a type of TCM used to deal with the pathogenic factor of “heat”). The study undertook clustering analysis using Discovery Studio software. The results were imported into Pajek software, and a network pharmacology method was used to build a drug–drug network according to structures and functions. The 3D structures related to coronary heart disease targets were imported, and the active ingredients in treating that disease were predicted and selected [25].

One study used a Taiwanese TCM database, the PubChem database, Gene database, and Ingenuity Pathway Analysis software to determine the molecular network of genes related to rheumatoid arthritis and network of the therapeutic effects of radix angelicae pubescentis. Following network analysis, the biological targets and pathways of *radix angelicae* for rheumatoid arthritis, such as aryl hydrocarbon receptor and histone H3, were visually presented [60]. Zhi-zi-da-huang (ZZDHD) decoction is a classic prescription in TCM and is applied in the treatment of alcoholic liver disease. In one study, the antioxidative mechanisms of ZZDHD in treating alcoholic liver disease were explored using molecular docking and network pharmacology. A drug–target network was established to predict the relationships between active compounds and targets. Multiple active compounds of ZZDHD were screened based on four potential targets (e.g., cytochrome P450 2E1) involved in alcohol-induced oxidative damage [50]. Complex system studies benefit considerably from network pharmacology and have indicated new methodologies. In one study, two interaction or association networks were identified using Cytoscape software, namely, the drug-herb (D-H) network and the drug–target (D-T) network. The interactions among compounds were used to construct the D-H network. The compounds were connected with their potential targets to construct the D-T network. The study used a combination of network pharmacology and pharmacokinetic analysis to examine the material basis of the bu-shen–zhuang-gu (BSZG) formula (a TCM). By means of the LC-MS/MS method and pharmacokinetic results, network pharmacology analysis indicated that four compounds appeared to be the material basis for BSZG [61]. One study closely integrated data related to protein–protein interaction, disease-related genes, and microarray experiments to establish an acute myocardial ischemia (AMI)-specific organism disturbed network (AMI-ODN). It then developed the network recovery index for organism disturbed network (NRI-ODN) to examine the properties of qi-shen-yi-qi (QSYQ) formula and its ingredients in restoring the disturbed AMI network. It was found that the whole QSYQ prescription, which had a high NRI-ODN score, showed a better performance than any single herb alone. At the same time, the two primary herbs used in QSYQ outperformed the supplementary herbs. AMI-ODN and NRI-ODN demonstrated that the different herbs in QSYQ play different roles in treating AMI [62].

A TCM network pharmacology platform has been constructed to determine in a systematic manner how herbal formulas adjust imbalances in the human body. One case study investigated the qing-luo-yin (QLY) formula, used for treating rheumatoid arthritis [63]. The detailed procedures were as follows:

First, after excluding the ingredients that appeared more than once, 235 available ingredients were collected from HerbBioMap and other literatures on the four herbs in QLY formula.

Second, drugCIPHER method, a self-developed regression model which can utilize and analyze FDA-approved drug structures, human protein–protein interactions, and drug–target interactions, was utilized to predict the target profile for each compound with known chemical structure from QLY.

Third, RA-related genes or proteins, which resulted from combining and compiling RA-causing genes from CIPHER and OMIM prediction and the target proteins of anti-RA drugs from DrugBank, were utilized as seeds to find their corresponding interacting proteins in the HPRD, and the results brought about an enlarged network as the RA-specific network. Comodule analysis was performed based on the network target to screen active compounds and synergistic drug combinations.

Fourth, Principal Component Analysis (PCA) was utilized to visually assess the characteristics of target profiles of each herb and its compounds, and the results showed that the target profiles can be roughly divided into three groups which can be used to cluster and identify active Ingredients in QLY.

Finally, the candidate targets of ingredients in each herb predicted by drugCIPHER were mapped into the RA-specific network to search active and synergistic ingredients, and an ingredient-ingredient interaction network was developed in terms of target proteins of each compound.

As a result, the active and synergistic compounds in QLY were predicted, and their combined role in the formula was interpreted. Two compounds from ku-shen (one of the main herbs in QLY)—matrine and kurarinone—appear to have a powerful synergistic effect, showing possible interactions with different compounds in the same herb. Some of these findings have been demonstrated in the literature; others need to be verified in future experiments [63]. In this study, targets, which possibly cause side effects, should also be collected to take part in the prediction. Then, if the predicted active or synergistic compounds induce serious side effects at the same time, then the chemical structure modifications of these compounds should be adopted or else abandonment will be a wise choice. Furthermore, dosage is undoubtedly crucial during the course of the drug predictions; however, the factor of dosage has not well been considered in the network pharmacology, possibly due to the technique reasons. Hopefully, when network pharmacology is used to study the effects of TMs, every compound’s dose should be combined wonderfully with other factors in the near future.

In the network pharmacology, the used computational methods are very important in the development of more specific drug combinations in TMs. Interestingly, in another study, DT-Web, a web-based application, was proposed to predict drug combination and drug–target interactions. DT-Web, which is a software resource accessible via web at DT-Hybrid, http://alpha.dmi.unict.it/dtweb/, integrates PathwayCommons, DrugBank, and DT-Hybrid algorithm to guide the drug combinations research by using a multi-target, multi-drug, multi-pathway approach. If the drug combination research of QLY formula is carried out using DT-Web system, then the possible procedures may be as follows.

At first, off-line database building pipeline should be implemented; and then, genes were retrieved from CIFER and OMIM prediction, and input into DT-Web system, and real-time prediction pipeline will be followed; and finally the list of relevant targets and their interacting drugs will be demonstrated. The predicted drug combinations from DT-Web and last platform of TM network pharmacology can be compared to serve proof or references.

Compound-target networks, main component-target networks, and natural product-target networks have been established using molecular docking. Literature retrieval and network analysis have been used to identify potential effective multiple components and their possible synergistic mechanisms. One case study examined bu-shen-huo-xue (BSHX) formula, which is frequently applied in treating chronic kidney disease. It was found that BSHX regulated a multi-pathway network (e.g., adjusting the fibrinolytic balance) to exert a therapeutic effect. Five compounds, such as tanshinone IIA and calycosin, may be the effective compounds in BSHX [64]. A network pharmacology method was used to predict the potential anti-diabetic ingredients in one TCM prescription—ge-gen-qin-lian decoction (GGQLD). The target profiles of all available GGQLD ingredients were clustered with anti-diabetic drugs approved by the US Food and Drug Administration (FDA). Among the active ingredients identified was 4-hydroxymephenytoin, a novel anti-diabetic ingredient from Puerariae lobata radix (ge-gen). At the same time, berberine in huang-lian (one of the herbs in GGQLD) was selected to search for possible synergistic effects with the 286 GGQLD ingredients using network pharmacology. Several characteristics of the molecular network, such as node importance and shortest path distance between two ingredient target profiles, were comprehensively used to quantify the synergistic effects. The results showed that berberine and guaifenesin in huang-qin might produce synergistic anti-diabetic effects [65].

Prediction of absorption, distribution, metabolism, and excretion as well as chemical analysis have been closely integrated with network pharmacology to explore active compounds and their underlying molecular mechanisms. Using UPLC-ESI-MS/MS, 48 compounds were identified from dragon’s blood tablets (a TCM formula). Interactions among these components or metabolites and their inferred targets were examined through constructed networks; a comparison was made of the known targets for colitis and the inferred targets [66]. Network pharmacology was used to study the druggability, molecular docking, and components of the huo-xiang-zheng-qi pill, which is used to treat functional dyspepsia (FD). It was found that the synergistic effects of 14 active ingredients, which had close interactions or a high network degree with certain special proteins, played a significant role in the treatment of FD [67]. Using a compound-target network constructed after literature mining, one study aimed to elucidate the multiple compounds, multi-target effect of xue-sai-tong injection for retinal vein occlusion. A compound-target network was established for anti-occlusion effects of the injection. Fifteen potential targets associated with inflammation, apoptosis, coagulation, and angiogenesis were found. Among them, IL-1β, VEGF, and IL-6 were further demonstrated experimentally [68].

Using a computational systems approach, one study constructed networks for multiple components, multiple targets, multiple pathways, drug-likeness prescreening, and molecular docking among 69 potential active ingredients of gan-fu-le (GFL). The active ingredients were identified using a network approach and were closely related to eight targets connected with hepatocellular carcinoma (HCC). Such important pathways in the treatment of HCC as PI3K-Akt and PI3K-Wnt were identified. GFL may be effective in such areas as cancer and infection [69]. One study constructed a network for components, targets, and diabetes related to *Corydalis yanhusuo* alkaloids using the CNKI, VIP, PubMed, and Wanfang databases; targets for diabetes treatment were determined with the OMIM database. Cytoscape was employed for analysis. Based on experimental evidence, the network results showed that *Corydalis yanhusuo* alkaloids were related with such genes as NOS3 and KCNJ11. This suggests the potential effects of *Corydalis yanhusuo* in diabetes and diabetic complications, especially with non-insulin-dependent diabetes [70]. Qi-gui-tong-feng tablets (QGTFT) are a TCM formula for treating gout. Based on molecular analysis, it was inferred that the flavonoids in QGTFT could be new potent drugs for gout. Network analysis identified dongbeinine, verticinone N-oxide, and peimine, which had a high degree of similarity to xanthine dehydrogenase, xanthine oxidase, and matrix metalloproteinase-9. When those targets are inhibited, inflammation by uric acid is reduced, the formation of uric acid is prevented, and the immune response is regulated. The molecular mechanisms of QGTFT were explored, and the research results will benefit the quality control and clinical application of QGTFT [53].

### 4.2. Network Pharmacology Research into TMs Using New Methods or Databases

One investigation developed a distance-based mutual information model (DMIM) to determine the relationships among the herbs used in numerous herbal formulas. DMIM is a powerful method to identify combinations of herbal formulas and has led to new discoveries. That study provided the first evidence for co-modules across multilayer networks possibly underlying the means of combining herbal formulas. A case study demonstrated that six herbs in the liu-wei-di-huang formula were associated with common responsive genes in neuroendocrine immune and cancer pathways [71]. The cardiovascular disease herbal database (CVDHD) was established to provide rich information about drug discovery from natural products. The CVDHD includes details of 35,230 molecules as well as their identification information and molecular properties. The database contains docking results among all the molecules and 2395 target proteins, clinical biomarkers, pathways and cardiovascular-related diseases. Drug and lead discovery from natural products and investigating mechanisms will be easier by combining the CVDHD with network pharmacology [72]. One report retrieved genes from the RGD public database and protein–protein interaction relationships from the HPRD and BioGRID databases. The data were integrated to create a cerebrovascular disease network. Then, the targets of the five main compounds in hong-hua were collected from PubMed. Regulation in such areas as neurogenesis, angiogenesis, and apoptosis was included among the main biological activities. It was found that quercetin and hydroxysafflor yellow A have synergistic, anti-apoptosis effects and express a cerebrovascular-protective activity. Network pharmacology revealed the multi-pathway and multiple-compound mode of hong-hua injections for treating cerebrovascular disease [73]. The concept of structural components of TCM has been introduced into TCM drug development; this involves the components being used in particular proportions and with clear mechanisms. In one study, network pharmacology was integrated with structural components of TCM to systematically return systematic pathology and biological networks to normal ranges through multiple targets, levels, and channels [74].

Analysis of gene expression and high-throughput screening makes gene expression profile technology a useful tool for mechanistic investigations and in many technical areas. In one case study, research into the active compound groups of bai-mai-san, which is a TCM formula, was combined with gene expression profiles and TCM network pharmacology [75]. One investigation collected the structures, biological activities, and screening results of 197,201 natural products. By exploring the chemical space using component analysis, the study found considerable overlap between FDA-approved drugs and natural products. This indicates that natural products have great potential as lead compounds. Polypharmacology has been greatly enriched by compounds with large degree and high betweenness centrality. The study employed virtual screening with high throughput: all-natural products were docked to 332 targets of FDA-approved drugs; based on a docking score-weighted model, predictions were made of potential ones for drug discovery [76]. In one study, a high-precision prediction network (integrating multiple molecular docking tools and two machine learning systems) was constructed to examine the binding possibilities of a protein and a compound. The two machine learning systems were applied to evaluate the binding modes and select the best one. The superiority of this approach was demonstrated in a case study and with other validations [77]. Rheidin A and C and sennoside C were first reported as multiple component drugs in treating type II diabetes [78]. Network target-based identification of multicomponent synergy algorithms was used to identify the synergistic effects of the multiple components in Chinese herbs. [79] The molecular mechanism of the multiple components, targets and pathways in tou-gu-xiao-tong capsules were revealed by analyzing the networks of the compound and drug targets [80,81]. Description of some TMs and relevant methodologies employed in network pharmacology is summarized in Table 1.

## 5. Discussion

Natural products, which give rise to many lead compounds, are of great importance in drug discovery [76]. Network pharmacology helps scientists analyze comprehensively the interactions among network parameters and potential drugs. It also allows examination of drugs’ pharmaceutical effects and molecular mechanisms. Thus, network pharmacology is a meaningful methodology in drug discovery [82]. Multi-target drug discovery based on molecular networks has gradually become accepted. The holistic concepts of syndrome differentiation and coordination in TM have aroused great interest in the field of drug discovery. TM, whose value has been tested in clinical practice, has a definite therapeutic effect, and it is less toxic in treating various diseases, especially complex chronic diseases, than Western medicines [83].

**Figure 3:**
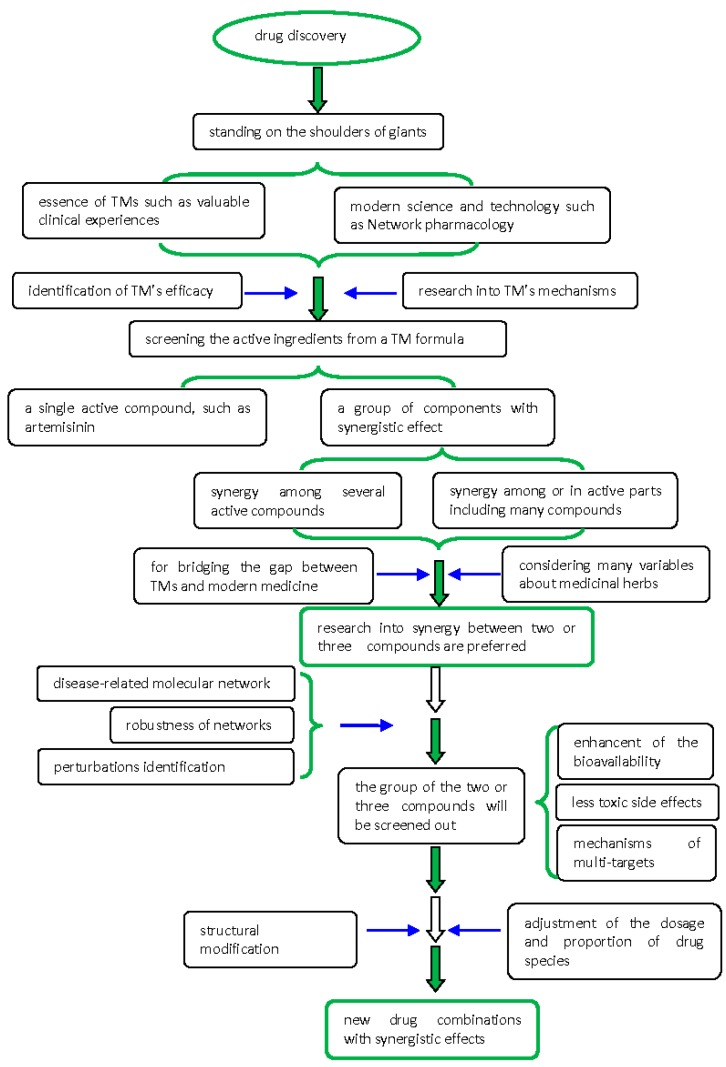
Drug discovery from traditional medicines (TMs).

The key to the problem with the modernization of TMs is the material foundation and action mechanism. A TM formula usually contains hundreds of ingredients. Identifying the active ingredients is the basis for understanding the mechanism of the whole formula. Of course, research into a single component, such as artemisinin, has achieved great success; however, in many cases, the effect of a single component is not ideal. The composition of formulas is an area of continuous exploration and involves accumulated synergy. A key issue in network pharmacology is evaluating the synergistic effect of multiple components and targets of TMs on the comprehensive influences of the disease-related molecular network. With medicinal herbs, there are many variables, including the texture of the materials used, different places of origin, and therapeutic stability. Thus, new drug development and research should first focus on the synergy among the monomer components. Here, new drug development can learn from the valuable experience of TM as well as using modern technology and ideas. Synergetic study of these compounds should be a bridge between Chinese and Western medicine; in this, network pharmacology should play an important role. In this regard, an important area of research is combined examination of chemical compositions of effective compounds, including the proportions used. When network pharmacology is applied to study the synergy of active components and various essential factors, targets in the network should be reliable and highly correlated with one another. The greater the reliability and correlation, the more believable will be the results of network research. Drug discovery should focus on the inherent robustness and perturbations identified in the disease-causing network rather than searching for individual disease-causing genes [84,85]. Discovery plans on new drugs with synergistic effects from TMs are summarized in Figure 3.

Researchers often use the active tracking method to isolate active compounds from TMs. However, in many cases, when purer substances are isolated, the drug actions are weaker or the side effects are stronger; this makes it difficult to identify the active substance. Network pharmacology research has revealed the network characteristics of drugs; it indicates that multiple drugs and targets may have better clinical efficacy and less toxic side effects than single target drugs. TMs, which have the synergistic characteristics of integrity and multiple components and targets, are certain to be the future direction for new drug research and development in the world. Network pharmacology observes the effect of drugs on disease from the network level; it reveals the secrets of complex drug synergism in the human body to identify more effective, lower toxicity multi-target drugs [58,86]. Early research on TM was limited to single effective compounds: the results showed that this approach was not the correct one for modernizing TM. Network biology analysis predicts that if in most cases, deletion of individual nodes has little effect on disease networks, modulating multiple proteins may be required to perturb robust phenotypes. Network biology analysis shows that, in many cases, disease networks cannot be affected by individual nodes. Thus, identification and proof of combined nodes in a biological network with synergistic effects will produce a desired therapeutic outcome [87,88]. There are so many ingredients in a TM formula or even a herb in it, among which some are have various chemical structures resulting in different interactions between drugs, targets and pathways; some have so little contents that they are often ignored by some methods. Topology-based pathway analysis thinks over a pathway’s topological structure, which promotes both sensitivity and specificity of the results [89]. Therefore, Topology-based pathway analysis has many advantages over other pathway analysis including IPA approaches in some cases.

With continuous development of network pharmacology research, research methods will also show varied characteristics. Research into the synergistic actions of TMs can begin in a higher position, effectively standing on the shoulders of giants. One of those shoulders is the essence of TM; the other is modern science and technology, such as network pharmacology. New drug strategies should be based on known effective prescriptions in TM. Such prescriptions provide a great many effective natural products for further research and reduce many potential side effects. Solid basic research should be conducted to identify the active ingredients—preferably in the same prescription or same plant. Then, based on all the active components, combinations of two or three monomers should be screened out using the network pharmacology method. Subsequently, through structural modifications and improvements as well as adjusting the dosage and proportions of the species used, the efficacy of the drug group will be continuously improved; side effects will be constantly reduced, such that, finally, the optimal synergistic drug combination will result. The longstanding, successful application of herbal formulas in TM makes it clear that phytotherapy has synergistic effects: these occur when the efficacy of a formula is greater than the summed effects of each individual ingredient. Various constituents in a formula or herb may enhance the bioavailability or function on several targets instead of one to produce the synergistic effects [16,71].

## Figures and Tables

**Table 1:** Summarized description of some traditional medicines (TMs) and relevant methodologies employed in network pharmacology.

TMs	Main Targets or Active Compounds	Employed Methodologies	Databases
*si-wu* decoction [59]	serine/threonine protein kinases	Cytoscape software	Kyoto Encyclopedia of Genes and Genomes pathway database, PharmMapper database
Radix angelicae pubescentis [60]	aryl hydrocarbon receptor, histone H3	Ingenuity Pathway Analysis software	Taiwanese TCM database, the PubChem database, Gene database
zhi-zi-da-huang decoction [50]	cytochrome P450 2E1, xanthine oxidase, etc.	Discovery Studio 2.5 Cytoscape 3.0.2	RCSB Protein Data Bank
bu-shen-zhuang-gu formula [61]	psoralen, psoralidin, isopsoralen, bergapten	Cytoscape 2.8.2	Entrez Gene, PubMed, CNKI
qing-luo-yin formula [63]	AKT1, PTK2, NF-κB	*Principal Component Analysis (PCA)*	HerbBioMap database, OMIM Morbid Map, DrugBank
*bu-shen-huo-xue* formula [64]	tanshinone IIA, calycosin	Cytoscape 2.8	Online Mendelian Inheritance in Man (OMIM), Genetic Association Database (GAD), Drugbank and Protein Data Bank
ge-gen-qin-lian decoction [65]	berberine and guaifenesin	drugCIPHER	Herb BioMap database, DAVID database
dragon’s blood tablets [66]	Hsp90, ADRB1, ADRB2	Therapeutic Targets Database 4.3.02, Navigator 2.2.1, Cytoscape 2.8.1	DrugBank, Human Annotated and Predicted Protein Interaction Database (HAPPI), Reactome, Online Predicted Human Interaction Database (OPHID), InAct, etc.
huo-xiang-zheng-qi pill [67]	14 compounds, 23 targets	Discovery Studio 3.0, Cytoscape 3.1.0	DrugBank, PDB database
*xue-sai-tong* injection [68]	IL-1β, VEGF, and IL-6	compound-target network	PubMed
gan-fu-le formula [69]	PI3K-Akt, mTOR, Wnt, Jak-STAT	Cytoscape 3.0.2, STRING 9.05	OMIM, RCSB protein Data Bank
*Corydalis yanhusuo* [70]	NOS3, KCNJ11	cytoscape 2.8.2.	OMIM, CNKI, VIP, PubMed, Wanfang databases
qi-gui-tong-feng tablets [53]	xanthine dehydrogenase, xanthine oxidase, etc.	DS 2.5	Therapeutic Target Database (TTD), RCSB
hong-hua injection [73]	Quercetin, hydroxysafflor yellow A	Cytoscape 3.1.0	PubMed, HPRD, BioGRID databases
